# Therapeutic Community Treatment of an Inmate Population with Substance Use Disorders: Post-Release Trends in Re-Arrest, Re-Incarceration, and Drug Misuse Relapse

**DOI:** 10.3390/ijerph120607059

**Published:** 2015-06-19

**Authors:** Alexandra Galassi, Elias Mpofu, James Athanasou

**Affiliations:** 1 Centre for Disability Research and Policy, University of Sydney, P.O. Box 170, NSW 1826, Australia; E-Mails: agal7796@uni.sydney.edu.au (A.G.); james.athanasou@sydney.edu.au (J.A.); 2 Discipline, Rehabilitation Counselling, University of Sydney, Lidcombe, NSW 2141, Australia

**Keywords:** community oriented treatment, inmates, re-offending, substance abuse, crime prevention

## Abstract

This systematic literature review maps the evidence for the effectiveness of the therapeutic community interventions (TCI) in reducing re-arrest, re-incarceration or drug misuse following release from prison, including the extent to which these effects are retained over time. The databases searched for the review included PsychINFO, Medline and Scopus and reference lists from relevant articles published between 2007 and 2014. Only quantitative studies that examined the effectiveness of TCI for a prisoner population with drug dependence at the time of initial incarceration were considered. Fourteen studies were identified for inclusion in the review. Three-quarters of the studies reported TCI were effective in reducing rates of re-incarceration. About 70% of studies that examined follow-up rates of drug misuse relapse found TCI effective in reducing rates of drug misuse amongst participants. TCI participation reduced re-arrests events in 55% of the studies. Results suggest TCI effective in the short-term rather than longer term for reducing rates of re-incarceration among participants, and to a slightly lesser extent, drug misuse relapse.

## 1. Background

Substance misuse disorders amongst criminal populations are common and hamper the potential for an individual’s successful reintegration into society after their release from prison [1]. This is of concern given that a large percentage of prison populations have substance misuse disorders [2]. For instance, over half of the inmates in state prisons in the USA reported experiencing symptoms that are consistent with a diagnosis of substance or drug misuse or dependence [3]. Once inmates are released from prison they are often likely to continue their involvement in drug use in the absence of support, and those who do are more likely to be re-arrested following release from prison than non-drug misusing peers [4,5]. Community oriented interventions that break this cycle and treat substance use disorders would lead to lower incidences of reoffending, re-arrest, re-incarceration, and drug misuse [6,7,8,9]. There are apparent public health benefits from ex-offenders not participating in drug misuse networks and maintaining healthy life-styles. Their maintenance of health and wellbeing is a benefit to family and community, directly from safer neighborhoods with reduced crime. Furthermore, the health support programs intended for ex-offenders also indirectly benefit other segments of the community sharing similar health risks or vulnerabilities as ex-offenders, making for improved public health overall. Therefore, it is important to consider the evidence for community oriented interventions that can best achieve this result. This study sought to map the evidence the effectiveness of therapeutic community intervention (TCI) programs in prison populations to prevent occurrences of re-arrest, reoffending, re-incarceration, and drug misuse relapse. 

### 1.1. TCI in Correctional Facilities

A variety of TCI are in use with prison populations [5,10]. They share the qualities that use a group based approach to rehabilitation, in which peers support and influence each other to develop pro-social behaviour and to work towards substance misuse recovery [5,11,12]. Increasingly, TCI for corrections may include an aftercare program or a transitional program, which reinforces new learning and assists ex-offenders to transition from correctional facilities to society [13]. They may also include drug-free wings in which inmates commit to not misuse substances for benefits such as more personal time and/or recreation [5]. Thus, TCI program qualities (e.g., length of program, presence of a compulsory aftercare program, presence of a non-compulsory aftercare program) would be important for their effect on crime and drug misuse reduction with prison populations. It is unclear from individual studies as to the extent to which TCI are effective to reduce the incidence of re-arrest, re-incarceration and substance misuse relapse in a cross section of ex-offenders [14,15,16]. Furthermore, some questions remain regarding the long-term impact of TCI treatment effects [15,16], and with or without an aftercare component [10,15,16]. Identification of TCI program qualities effective in reducing re-arrest, re-incarceration and drug misuse relapse is important to their adoption and implementation with prison populations [5,17]. 

### 1.2. Objectives 

This mapping systematic review aimed to scope the evidence on the potential for TCI to reduce criminal activity and drug misuse behaviours after release from prison, and the indicators of TCI programs associated with particular outcomes. The specific objectives of the review are to:

1. Trend the evidence on TCI in reducing drug misuse relapse, re-incarceration, and re-arrest among ex-offenders with substance abuse history.
2. Identify TCI qualities with preliminary evidence for reducing rates of reoffending and supporting sustainable drug misuse recovery.

A mapping systematic review has the limited objective to apply content analysis to summary the evidence for the purpose to suggest emerging findings that could guide future research on related practices. Ultimately, those from prison with drug dependence history do have the right to quality health care as other community members, regardless of their conviction [5,18].

## 2. Method

### 2.1. Search Strategy for Identification of Studies 

Four electronic research databases were searched: PsychINFO, Medline, Scopus, and Web of Science. These databases were chosen for their psychosocial relevance. They were searched using words that pertain to drug rehabilitation with TCI in an inmate population group. The terms ‘Drug Dependence’, ‘Therapeutic Community’, and ‘Prison’ were identified as key search terms and searches were restricted to a prisoner population. The key words relating to these terms differed slightly depending on which database was used. In each database the search was restricted to articles that were published since 2007 when the last review was conducted [5]. Articles were restricted to those written in English and published in peer reviewed journals. PsychINFO was searched using the subject headings: Therapeutic Community and (Prisoners or Criminals or Prisons) and (Drug Addiction or Drug Dependency or Drug Abuse). This search with aforementioned restrictions returned 16 results. Medline was searched using the subject headings: Prisoners and Therapeutic Community and Substance-Related Disorders. This returned nine results. Scopus was searched using the keywords: Therapeutic Community, and Substance Use and Prisoners. This returned 14 results. Web of Science was searched using the topic names: Therapeutic Communities and Substance Abuse and Prison. This returned 44 results. The results of the three searches were combined and duplicates were removed, resulting in a total of 60 articles. 

Articles that did not specifically concern the effectiveness of TCI and that were not empirical studies were then removed. Only those that concerned the outcome measures of rates of relapse, re-incarceration and recidivism were included. This process left a total of 13 articles. The reference lists of these articles were also examined for the purpose of procuring other relevant articles. Two additional articles were selected from these reference lists, which resulted in 15 articles remaining for consideration for inclusion in the systematic review. These studies were then read in full and one was eliminated as it was simply a re-analysis of results of one of the other selected studies. This resulted in a final sample of 14 articles.

### 2.2. Data Extraction and Management 

Studies for the analysis were selected if they met five of seven inclusion criteria proposed by Franche, Cullen, Clarke, Irvin, Sinclair, and Frank [19]: (a) the source population is clearly identified; (b) inclusion and exclusion criteria are appropriate (*i.e.*, appropriately assessed for drug dependence); (c) loss to follow up is less than 50 per cent of the original sample; (d) interventions are sufficiently described for replication; and (e) the outcome is defined and measurable. All of the 14 articles met these criteria as they scored five or more on this quality appraisal.

Table 1 presents the descriptive summary of studies included in this content analysis review. The TCI implemented in each study was further investigated with specific attention paid to whether the TCI was delivered traditionally or with modifications; whether an aftercare component existed, and if so, whether it was compulsory; the period of time until the follow up results were gathered; the outcome measures; and the interaction between these factors. 

## 3. Results

The findings from the present analysis suggest TCI is effective in reducing re-incarceration and drug misuse relapse as compared to alternate programs. This review has found that re-incarceration was the most effectively reduced outcome measure with both regular TCIs and with modified TCIs, with the vast majority of studies (75%) that examined this measure finding a reduction in re-incarceration rates. Moreover, re-incarceration was reduced in the longer term studies. Drug misuse relapse rates appear to be reduced for TCI participants (70% of studies examining relapse found positive results). Re-arrest was found to be reduced somewhat with 55% of studies examining re-arrest finding positive results. Furthermore, the results of long-term studies suggest poor prognosis for the two outcome measures of re-arrest and drug misuse relapse. With regards to treatment type, there appeared no relationship between program effectiveness and TCI program type (*i.e.*, modified TCI *vs.* regular TCI). Finally, participation in aftercare was seen to predict positive outcomes, even when aftercare was randomly assigned. 

### Outcomes Based on the Objectives of This Review 

Three dominant outcome variables were identified among the selected articles: (a) re-arrest, (b) re-incarceration rates, and (c) drug misuse (all measured at the time of follow-up). Eight of the studies examined more than one of these. Twelve of the selected studies measured the rates of re-incarceration, nine measured rates of re-arrest, and ten examined rates of drug misuse post release from prison. As each study examined a different set of outcome measures, each of the three outcomes of interest are reported on individually across all 14 studies. Detailed below are the major results relating to the present study’s research questions.

*Re-arrest*. Of the nine studies concerned with re-arrest, five supported the value of TCIs in reducing rates of re-arrest or time until first arrest [20,21,22,23,24]. However, the studies which were supportive of the effectiveness of TCI tended to be those that were conducted after a relatively short follow-up period (two years or less). Those studies with longer follow-up periods were less likely to be supportive of the effectiveness of TCI. 

**Table 1:** Descriptive Characteristics.

Citation	Country, State, and Setting	Method	Sample	Treatment Condition—Independent Variables	Follow up	Outcome Measures	Relevant Findings
Jensen & Kane (2010) [20]	Idaho, USA	Survival Analysis	1396 drug dependent offenders released from 4 Idaho prisons	TCI	2 years	Time until re-arrest post release from prison	Completion of a TCI had significant effect on delaying time until first re-arrest.
Jensen & Kane (2012) [25]	Idaho, USA	Survival Analysis	725 drug dependent offenders released from 4 Idaho prisons	TCI	4 years	Time until re-arrest post release from prison	Completion of TC did not have effect on reducing re-arrest.
Wexler & Prendergast (2010) [21]	Thailand	Longitudinal Study	769 drug dependent ex-residents in treatment programs—10.5% of whom were residents of 5 prison operated programs	TCI model implementation fidelity, prevalence of model modification, length in the program.	Average of 6 months after treatment	Change in criminal behaviour, re-arrest, drug abuse.	All outcomes reduced 6 months post treatment.
Lemieux *et al.* (2012) [26]	Southern State of USA. 3 Institutions.	Cross-sectional descriptive study.	226 drug dependent male and female youths released from three institutions in a Southern State after participating in a TCI. Follow up data available for 186 participants.	TCI model was used in prison for drug dependent youths.	2 years post release.	Recidivism—return to custody during the 2 year post release period.	10.3% of TCI participants were recidivists. Female ex-offenders were less likely to experience re-incarceration compared to males.
Messina *et al.* (2010) [27]	California, USA. Valley State Prison for Women.	Randomized experimental study, Longitudinal	115 drug dependent women ex-residents.	Gender responsive treatment model of TCI *vs.* standard prison based therapeutic community.	6 months and 12 months post release from prison	Psychological well-being, drug use post release, length of time in aftercare (based on completion of TCI), re-incarceration rates.	A gender sensitive TCI had greater reductions in drug misuse relapse, re-incarceration.
Miller & Miller,(2011) [28]	South Carolina, USA. South Carolina Department of Corrections.	Quasi-experimental, Longitudinal	303 first time, non-violent, drug-dependent youthful male ex-residents.	Modified TCI with a cognitive behavioural change component.	12 month follow up period	Recidivism (re-arrest), relapse (drug use), and parole revocation.	No difference between treatment and control group on any of the outcome measures.
Sacks, McKendrick & Hamilton (2012) [22]	Colorado, USA. Denver Women’s Correctional Facility	Randomised Clinical Trial	468 female ex-offenders with substance use disorders. 235 participated in TCI. 192 participated in cognitive behavioural intervention.	TCI treatment *vs.* Cognitive behavioural therapy. Voluntary TCI aftercare	6 and 12 months post release from prison	Outcomes across 5 domains—crime (re-incarceration and re-arrest), drug use, mental health, trauma, and HIV-risk behaviour.	TCI was more effective than cognitive behavioural therapy in reducing rates of re-arrest, drug misuse, and re-incarceration
Sas *et al.* (2008) [24]	Colorado, USA. Denver Women’s Facility	Randomised Clinical Trial	314 Females with substance use disorders. 163 participated in TCI, 151 in regular.	Experimental condition: participation in modified TC for female offenders. Control: CBT treatment	6 months post release from prison.	Mental health, Substance Use, Criminal Behaviour (re-incarceration and re-arrest), HIV risk.	Drug misuse rates reduced for both TCI and CBT interventions groups (no significant difference between two groups). Re-offending was lower with for TCI as compared to CBT group.
Sacks *et al.* (2012) [29]	Colorado, USA. 9 Colorado prisons.	Randomised trial	127 Male ex-offenders with co-occurring substance use disorders and mental disorders.	Men participated in either modified TCI program in Prison or standard care. Random assignment to either TCI aftercare (*n* = 71), or standard parole supervision & case management (*n* = 56).	12 months post release.	Re-incarceration and drug misuse relapse.	TCI with aftercare group had lower rates of re-incarceration and drug misuse relapse.
Sullivan *et al.* (2007) [30]	Colorado, USA. Colorado Department of Corrections.	Randomised Trial	139 Male offenders with substance use disorders and at least one co-occurring mental disorder.	Modified TCI (for a population with co-occurring mental disorder) (*n* = 75) CBT based treatment (*n* = 64). 44 TCI participants opted for 6 months of residential aftercare.	12 months post release.	Substance abuse and re-incarceration.	TCI had significant lower substance misuse. TCI had significantly lower illegal drug misuse. TCI had lower prevalence of re-incarceration. No separate analysis of the specific effect of aftercare.
Welsh (2007) [23]	Pennsylvania, USA. Five state prisons in Pennsylvania.	Longitudinal, quasi-experimental study	708 male ex-offenders with substance use disorders.	217 men participated in TCI programs in five state prisons. 491 men had access to substance abuse treatment only programs in prison.	2 years post release	Re-incarceration, Re-arrest, Drug abuse relapse.	TCI significantly reduced re-arrest and re-incarceration rates but did not reduce drug misuse relapse rates.
Welsh & Zajac (2013) [31]	Pennsylvania, USA. Five state prisons in Pennsylvania	Longitudinal, quasi experimental study.	1553 male ex-offenders with substance use disorders.	TCI programs in five state prisons (*n* = 555). Substance abuse treatment only programs in prison (*n* = 998).	4 years post release	Re-incarceration, Re-arrest, Drug abuse relapse.	TCI resulted in significantly reduced probability of re-incarceration. TCI failed to significantly reduce re-arrest or drug misuse.
Welsh, Zajac & Bucklen (2014) [32]	Pennsylvania, USA. State Correctional Institution at Chester.	Longitudinal quasi-experimental design.	604 male ex-offenders who participated in drug treatment in prison. Participants had no other serious mental health issues.	TCI (*n* = 286). Substance abuse group counselling program (*n* = 318).	3 year follow up	Rates of re-incarceration 3 years after release from prison.	There was no significant difference in re-incarceration rates by treatment modality. Treatment completion rather than modality was a significant predictor of re-incarceration.
Zhang, Roberts & McCollister (2011) [33]	California, USA.	Longitudinal quasi-experimental	798 male ex-offenders with substance abuse problems at the time of initial incarceration.	TCI (*n* = 395), some with aftercare (*n* = 101), while others did not (*n* = 294). No treatment (*n* = 394).	1 year follow up and 5 years follow up	Re-incarceration and re-arrest 1 year post release.	TCI Aftercare participants less likely to be re-incarcerated (not statistically significant). TCI re-incarceration rates equivalent to no treatment. TCI with aftercare significantly fewer days in prison than those without aftercare. No differences in re-arrest rates or re-incarceration

With regard to the relationship between the nature of the program and subsequent rates of re-arrest, there appear to be no systematic effects. There was an even distribution of modified TCIs and regular TCIs across all studies (both those that supported the reduction of re-arrest rates, and those that did not).

*Re-incarceration.* All but two of the seven studies that examined the outcome measure of re-incarceration supported the effectiveness of TCI. Five of seven studies [22,23,24,30,31] found that TCI was no more effective than no in prison treatment in reducing re-incarceration. However, in this study there was a mandatory after-care component for all participants, suggesting that aftercare may have been the decisive factor in reducing rates of re-incarceration. The impact of aftercare in reducing rates of re-arrest was also discussed in five more of the twelve studies that examined rates of re-incarceration. The two studies with contradictory findings [32,33] concluded that TCI was only marginally more effective in reducing incarceration than an alternate, less intensive substance misuse treatment.

*Drug misuse relapse.* The value of TCIs in reducing drug misuse relapse rates was observed in seven of the nine studies that examined this outcome. Of these six studies, four studies included an aftercare program that could have contributed to the positive results of the TCI [22,27,29,30], and three used a modified TCI [24,27,30]. 

*TCI model type.* Of the fourteen studies, seven studies analysed the effect of a regular TCI program, while seven looked at results of a modified therapeutic community with a cognitive-behavioural therapy component. Two of the seven studies concerned with a regular TCI found consistently positive results on all the outcomes of interest that were measured [20,22]. Two of the studies found that TCI was no more effective than the alternate treatment on any measure [25,32]. One study [28] found that the regular TC only reduced re-incarceration rate and not re-arrest incidence or drug misuse relapse (when compared with alternate treatment) [31]. However, one study [23] found that TCI effective in reducing re-arrest and re-incarceration and not drug misuse (when compared with alternate treatment). Finally, one study [33] reported TCI was more effective in reducing re-incarceration (but no more effective than alternate treatment in reducing re-arrest). 

Of five studies that investigated the effects of a modified TCI with a cognitive behavioural component, three reported positive effects across all outcomes measured [21,27,30]. One study [28] found modified TCI was no more effective than alternate treatment in reducing re-arrest and re-incarceration (but not drug misuse relapse) [24,26]. Finally, one study [29] found modified effectiveness in reducing drug misuse relapse (not criminal outcomes) [29].

Six of the 14 studies included some form of aftercare program [22,27,29,30,32,33]. Those studies that included an aftercare program were found to be more effective than those that did not, with five of the six suggesting that aftercare reduced one or more of the three outcome measures [22,24,27,29,33]. For the study in which no positive results were observed across any of the outcome measures, the aftercare element was mandatory for all participants [32]. Only one study examined results from a random allocation to an aftercare or standard parole [29] (as opposed to voluntary participation), and found that the aftercare group was less likely to be re-incarcerated. 

*Long-term effects*. Four of the 14 studies examined long-term follow up results (greater than 2 years’ follow-up) [25,31,32,33]. Two of the studies examined results at four years’ follow-up [25,31], one at three years’ [32] and one examined outcomes at five years’ follow-up [33]. Of the three outcome measures, TCI participation was only associated with lowers rates of re-incarceration in these long-term follow-up results [31,32,33].

## 4. Discussion

The evidence from this scoping review provides support for the TCI in reducing severe criminal reoffending for a drug misusing population who participate in such a program. Specifically TCI with after care appear more effective in reducing the rates of re-incarceration and drug misuse relapse compared to alternative programs. Prior literature has also given support to the positive effects of TCI on recidivism and drug misuse relapse prevention [5,34]. TCI with aftercare appear effective in to supporting participants to learn to adjust to life outside of prison. They likely achieve this effect by helping participants to learn life skills and coping strategies important to community living post-release [35]. 

The effect of aftercare is encouraging to public health oriented interventions with ex-offenders with substance abuse histories; this is particularly the case since after care programs make a positive difference to reducing drug misuse-related re-offending. However, it must be noted that in some studies included in this review the aftercare program participation was voluntary, and that aftercare *versus* no aftercare was not randomly assigned. This finding suggests that participant motivation to engage aftercare likely mediates its positive effects on reducing re-incarceration, drug misuse relapse and re-arrest post release. The likely influence of participant motivation on TCI with aftercare effects on the study outcomes is further supported by the fact that the only study that did not produce positive aftercare results involved mandatory aftercare [32], in which intervention effects may have been masked by regression of scores towards the mean. In this study some participants who participated in aftercare may not have been motivated to do so, and which would reduce the observed magnitude of the TCI treatment effect.

Re-arrest rates appear to be relatively unaffected by participation in a TCI. This finding may be explained by the fact that the probability of re-arrest is higher in ex-offenders compared to the general population although the re-arrest might not necessarily lead to conviction or be drug misuse related. Thus, although participants of TCI are less likely to engage in serious criminal activity (which would make re-incarceration likely) they may experience re-arrest for a variety of misdemeanours. Therefore, the evidence from this mapping systematic review suggests that TCI may not necessarily reduce overall rate of criminal activity among ex-offenders;—although effective in reducing offense severity which would lead to re-incarceration. 

### Limitations of the Overall Completeness and Applicability of Evidence

First, the findings from this scoping review chart trends in the evidence for TCI in reducing the rates of re-incarceration, drug misuse relapse and re-arrest among ex-offenders with substance misuse history. The study selection criteria by research design were deliberately inclusive allowing for evidence from a variety of investigations. Findings from a scoping review are best only indicative rather than conclusive. Only a few studies are as yet available for review. As additional studies are available, future review studies could examine more closely the evidence for specific TCI program effects on expected outcomes controlling for study design. Second, a sampling limitation of the studies from this review is that most were largely on the US prisoner settings. Only one study examined TC evidence from outside of the USA [21], this was set in Thailand. The extent to which TC programs are used in the US, is unknown, and this review covered by the review only represent six states (of 50), and potentially only 16 prisons (of thousands).Therefore, there is a valid question about the extent to which the findings of this study are applicable to prisoner populations across the USA and around the world. 

Third, not all studies examined all three outcome measures (re-incarceration, re-arrest, and drug misuse relapse) and the evidence was more prevalent for some outcomes (*i.e.*, re-incarceration) than others (*i.e.*, re-arrest). This further underscores the fact that the comparative outcomes data reported findings reported here are quite tentative and should be treated with caution. 

A fourth limitation is that the grey or unpublished literature was not considered in this review. This might have excluded studies which would provide a more complete picture of the efficacy of TCI intervention to prevent re-offending and related outcomes.

## 5. Summary and Conclusions

Substance misuse disorders are a mental health issue within the prison system. This evidence aggregated for this study were from a range of prison populations including both men and women with co-occurring mental disorders, and other general prison populations. Upon descriptive summary analysis, the evidence points to several dominant findings. With regard to the first research objective, it appears that the TCI, regardless of program type, fare better than alternative treatment methods in reducing rates of re-incarceration, drug misuse relapse, and re-arrest than other treatment alternatives. TCI treatment produced the greatest effect on reducing re-incarceration and drug misuse relapse rates (irrespective of aftercare). More specifically, those TCI programs that are and coupled with an aftercare program may be more effective in reducing re-incarceration and drug misuse relapse than alternative programs. Results of this review suggest that TCI comparatively more effective in long-term reduction of rates of re-incarceration rather than drug misuse relapse or re-arrest. 

The review highlights the potential value of TCI for a population of drug misusing offenders in reducing rates of re-incarceration in particular, as well as drug misuse relapse, and to a lesser extent, re-arrest. It supports the importance and benefits of tackling the problem of drug dependence in a prison setting and intervening in the vicious cycle of drug misuse and incarceration. To further bolster the confidence with which the positive effects of TC observed can be help, data from extended follow-up of inmates are required. 
